# Supplementary material for: Designing Sustainable Polymer Blends: Tailoring Mechanical Properties and Degradation Behaviour in PHB/PLA/PCL Blends in a Seawater Environment
Source: Polymers (Basel). 2023 Jun 29;15(13):2874. doi: 10.3390/polym15132874 (PMC10347181; doi:10.3390/polym15132874)
Supplement: Supplementary file 1 [file polymers-15-02874-s001.zip › polymers-2421721-supplementary.pdf]

Supplementary

Designing Sustainable Polymer Blends: Tailoring Mechanical Properties and Degradation Behaviour in PHB/PLA/PCL Blends in a Seawater Environment

Leonardo G. Engler [1,2], Naiara C. Farias [1], Janaina S. Crespo [1,2], Noel M. Gately [3], Ian Major [1], Romina Pezzoli [3] and Declan M. Devine [1,*]

[1] PRISM Research Institute, Technological University of the Shannon: Midlands Midwest, Athlone Campus, University Road, N37 HD68 Athlone, Ireland; a00278634@student.tus.ie (L.G.E.); a00203999@student.tus.ie (N.C.F.); jscrespo@ucs.br (J.S.C.); imajor@ait.ie (I.M.)

[2] Postgraduate Program in Materials Science and Engineering, University of Caxias do Sul, Francisco Getúlio Vargas Street, 1130, Caxias do Sul 95070-560, Brazil

[3] Applied Polymer Technologies Gateway, Technological University of the Shannon: Midlands Midwest, Athlone Campus, University Road, N37 HD68 Athlone, Ireland; n.gately@ait.ie (N.M.G.); rpezzoli@ait.ie (R.P.)

* Correspondence: ddevine@ait.ie

Mechanical prperties of the blends - Seawater degradation

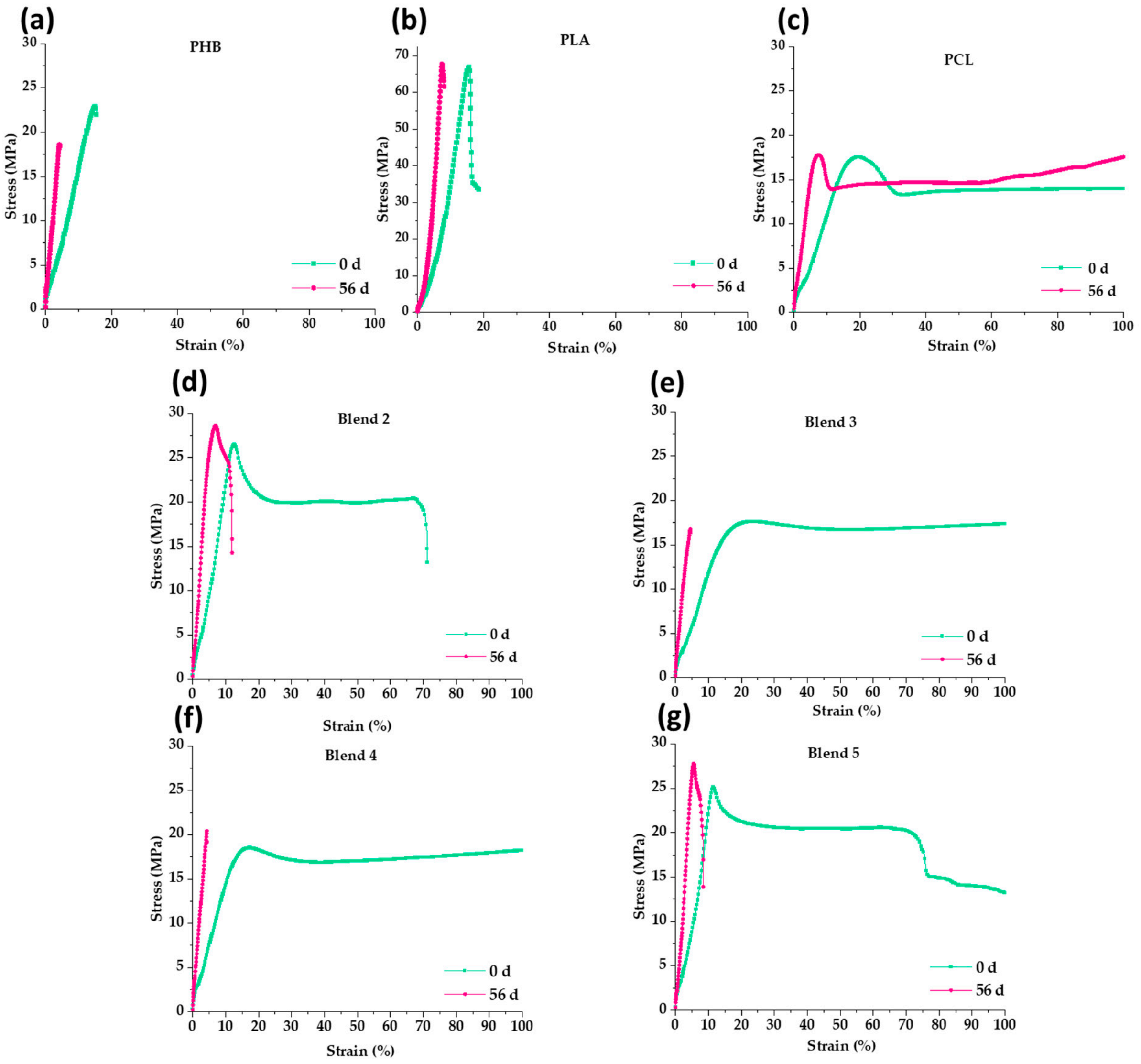

Figure S1. Stress vs Strain of neat PHB/PLA/PCL and their respective blends at initial state and after 56 days of seawater degradation: (**a**) PHB, (**b**) PLA, (**c**) PCL, (**d**) 50/50/0, (**e**) 50/0/50, (**f**) 50/25/25, and (**g**) 30/50/20 (wt. % PHB/PLA/PCL).

Fourier-transform infrared spectroscopy (FTIR) - Seawater degradation

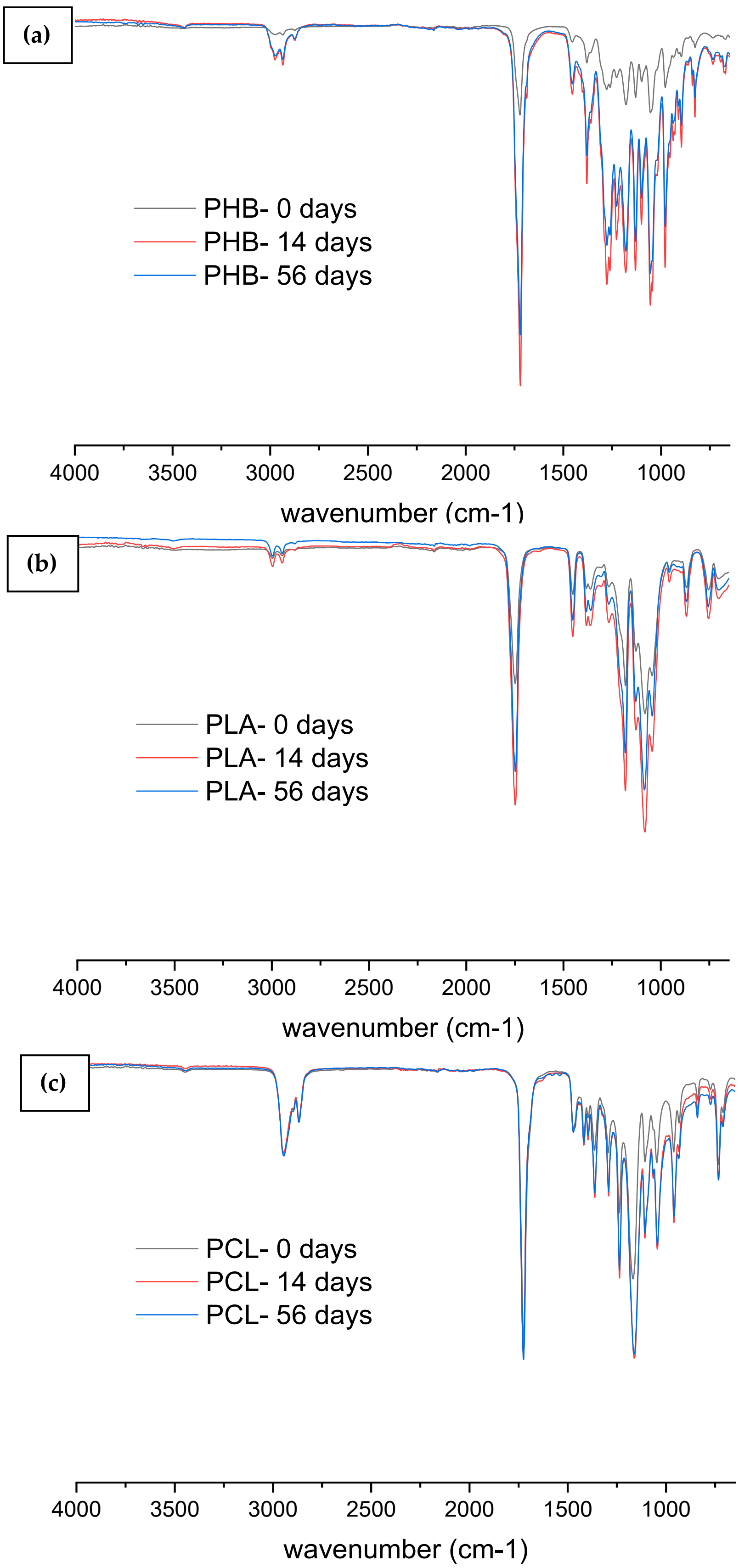

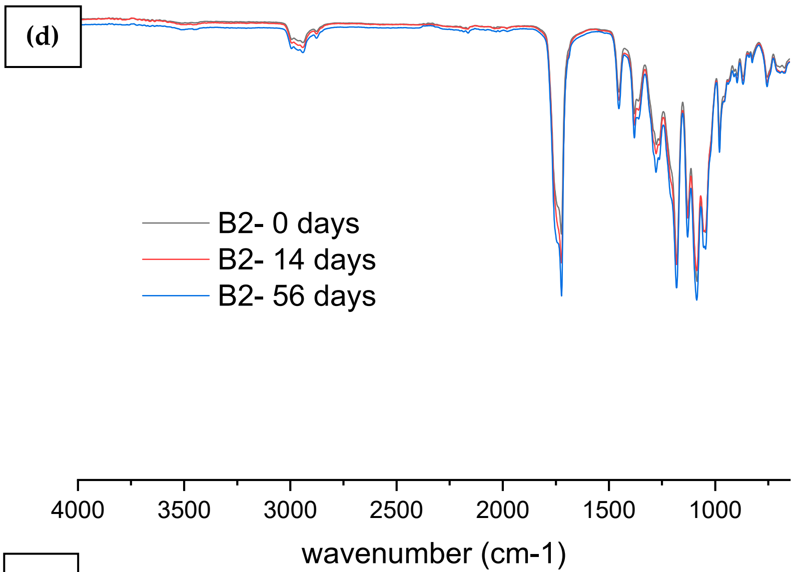
(d)
B2- 0 days
B2- 14 days
B2- 56 days
4000
3500
3000
2500
2000
1500
1000
wavenumber (cm-1)

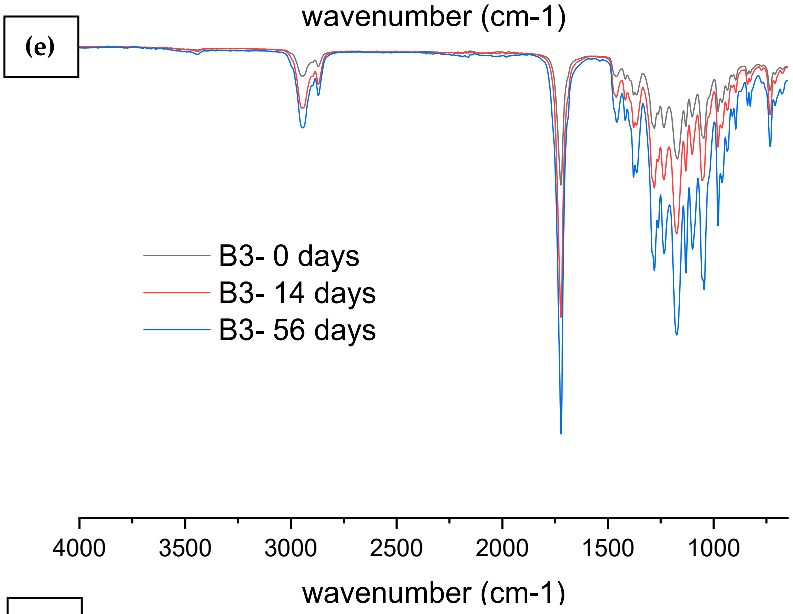
(e)
B3- 0 days
B3- 14 days
B3- 56 days
4000
3500
3000
2500
2000
1500
1000
wavenumber (cm-1)

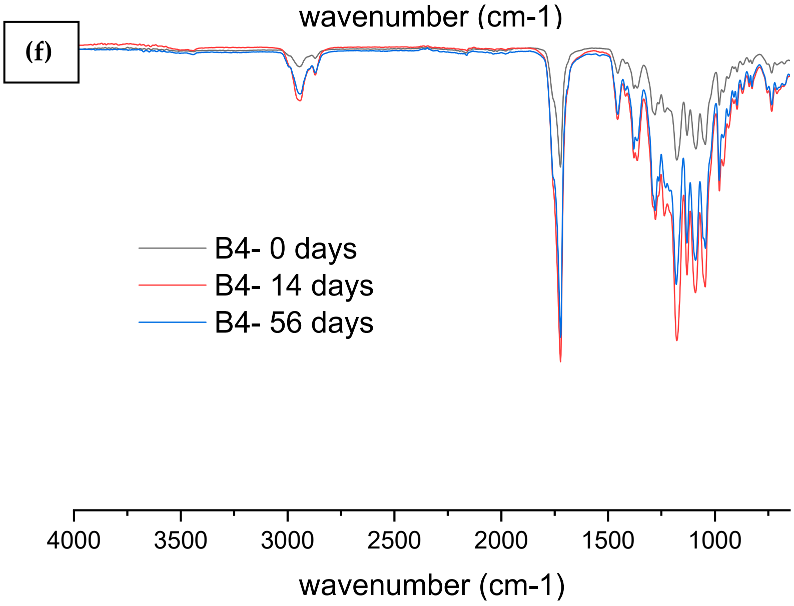
(f)
B4- 0 days
B4- 14 days
B4- 56 days
4000
3500
3000
2500
2000
1500
1000
wavenumber (cm-1)

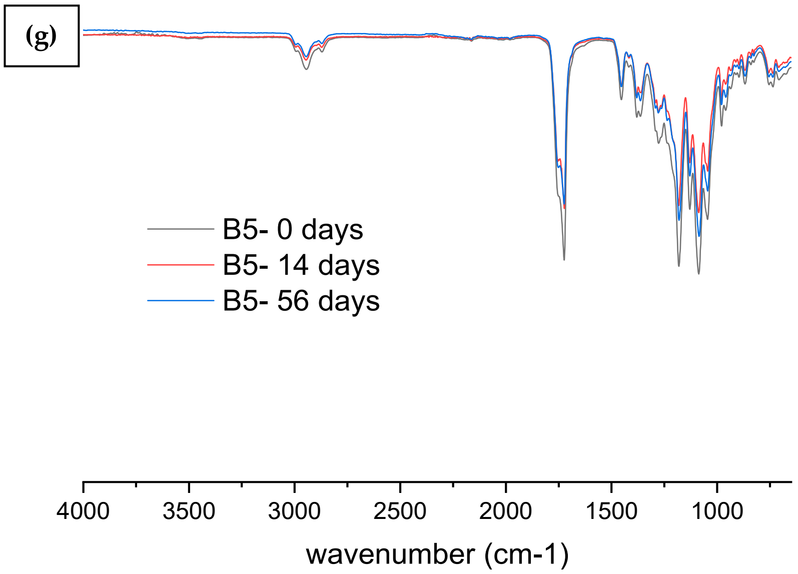

Figure S2. FTIR of neat polymers PHB/PLA/PCL and their respective blends: (**a**) PHB, (**b**) PLA, (**c**) PCL, (**d**) B2: 50/50/0, (**e**) B3: 50/0/50, (**f**) B4: 50/25/25, and (**g**) B5: 30/50/20 (wt. % PHB/PLA/PCL).

Thermogravimetry (TG) and DTG analysis

(a)

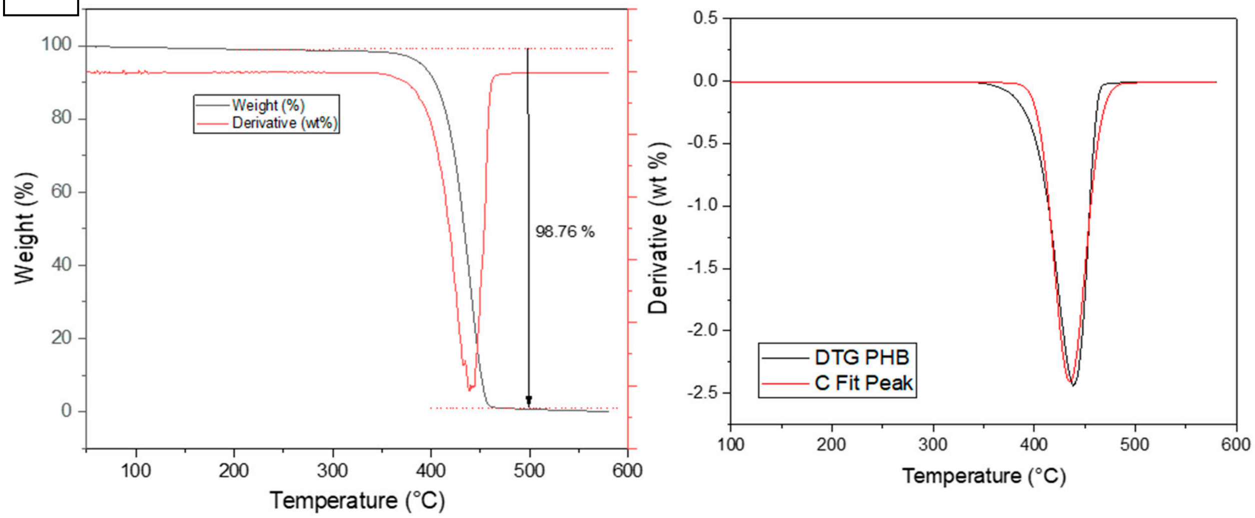

(b)

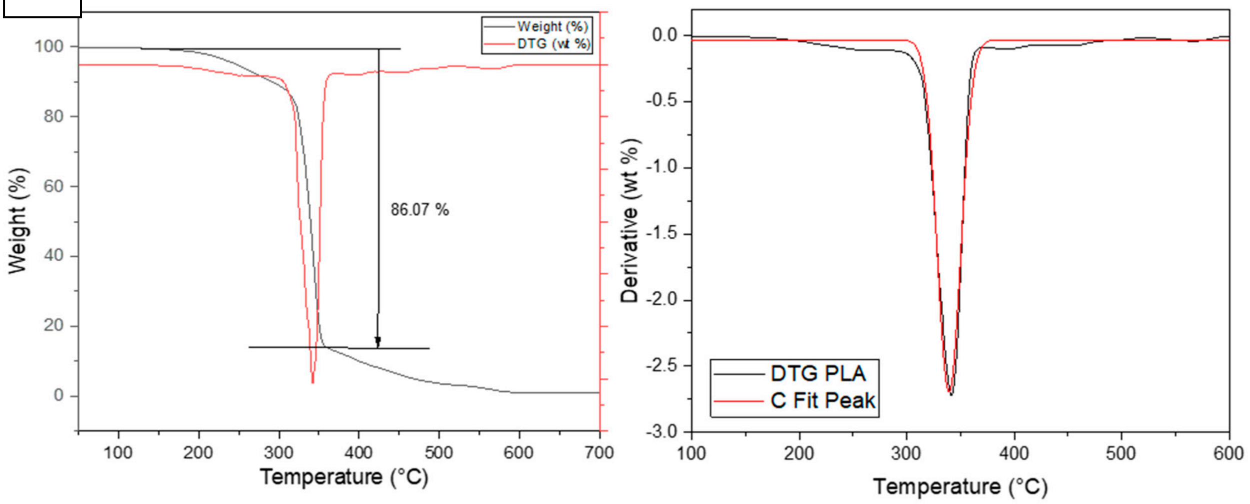

(c)

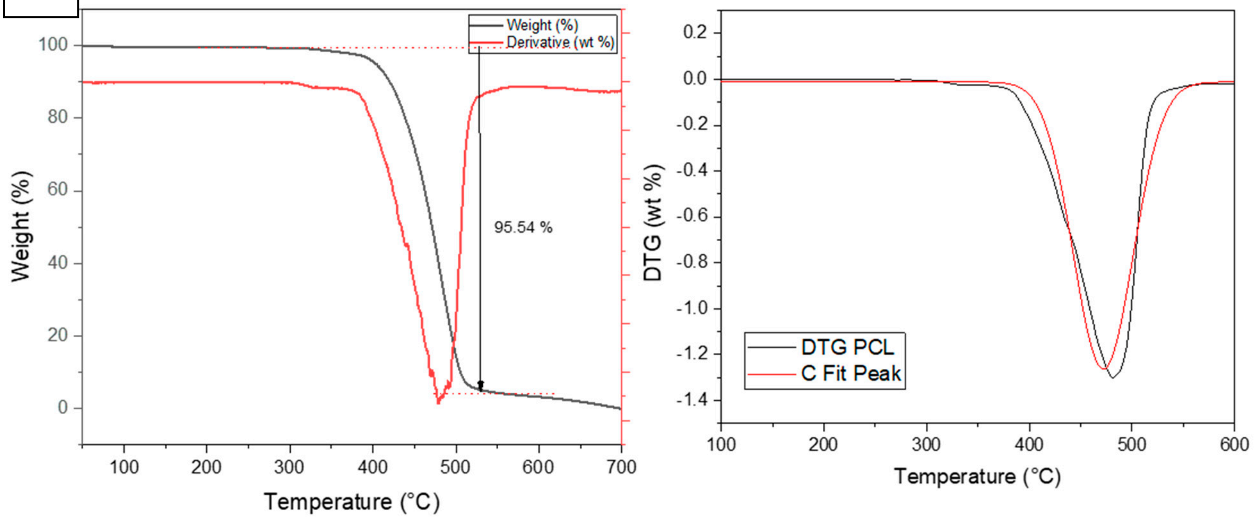

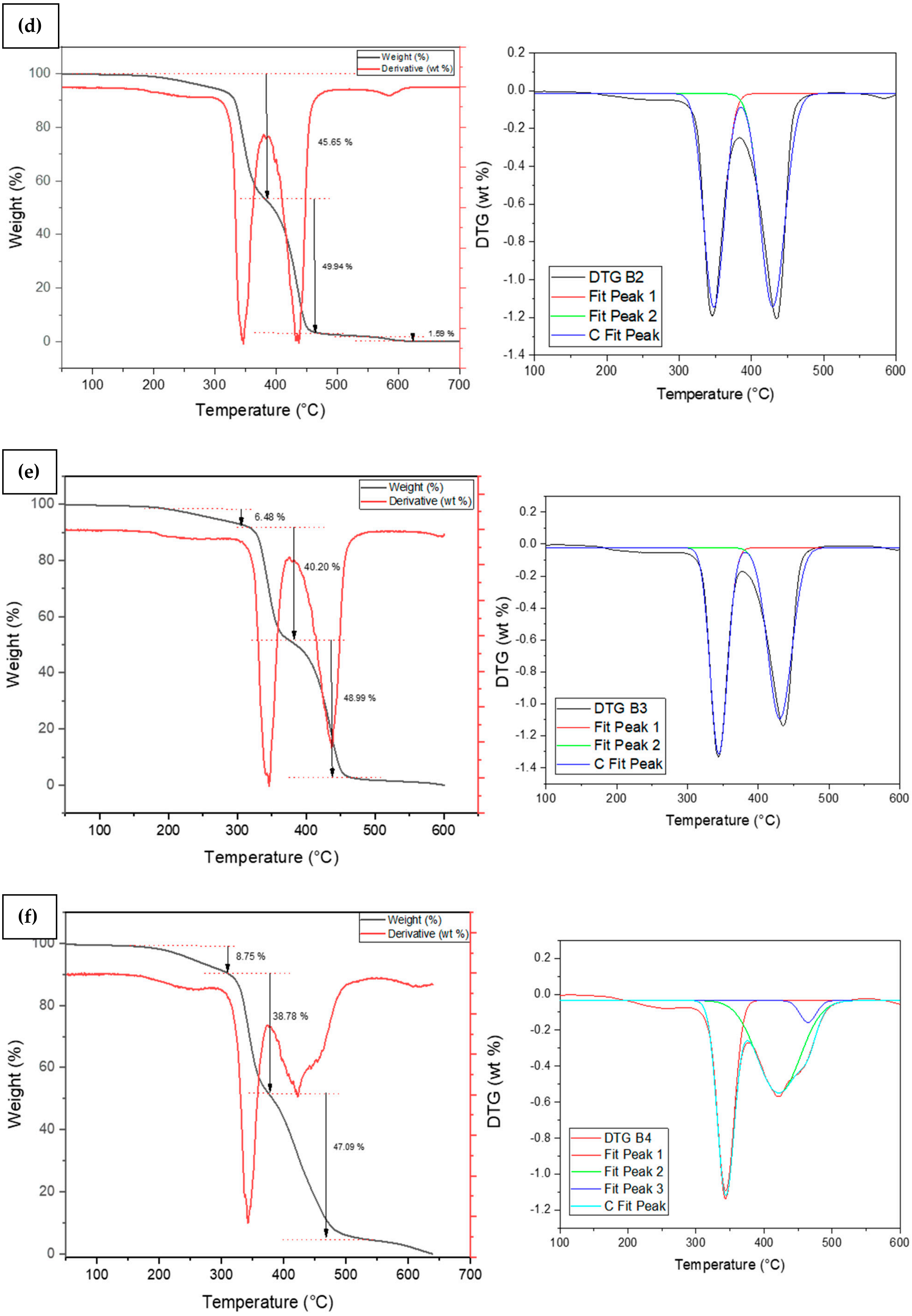

(d)
Weight (%)
Derivative (wt %)
45.65 %
49.94 %
1.59 %
Temperature (°C)
DTG (wt %)
DTG B2
Fit Peak 1
Fit Peak 2
C Fit Peak
(e)
6.48 %
40.20 %
48.99 %
DTG B3
(f)
8.75 %
38.78 %
47.09 %
DTG B4
Fit Peak 3

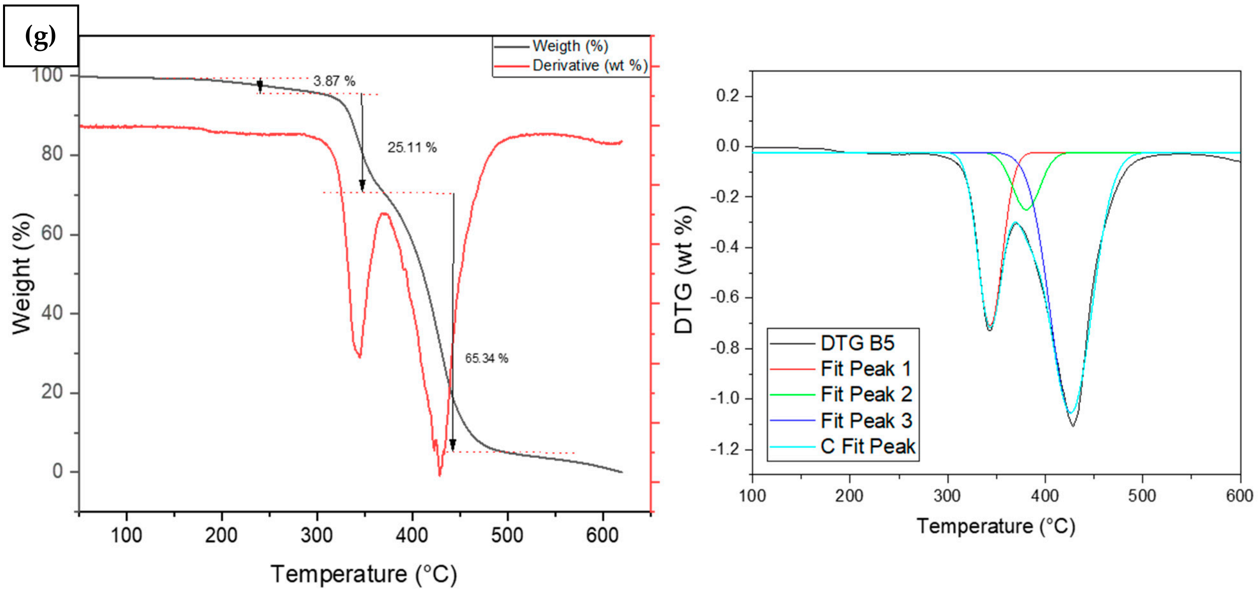

Figure S3. TGA and DTG of the neat polymers PHB/PLA/PCL and their respective blends (**left:** TG and DTG/**right:** deconvolution of the DTG peaks): (**a**) PHB, (**b**) PLA, (**c**) PCL, (**d**) B2: 50/50/0, (**e**) B3: 50/0/50, (**f**) B4: 50/25/25, and (**g**) B5: 30/50/20 (wt. % PHB/PLA/PCL).

Differential scanning calorimetry (DSC) - Seawater degradation

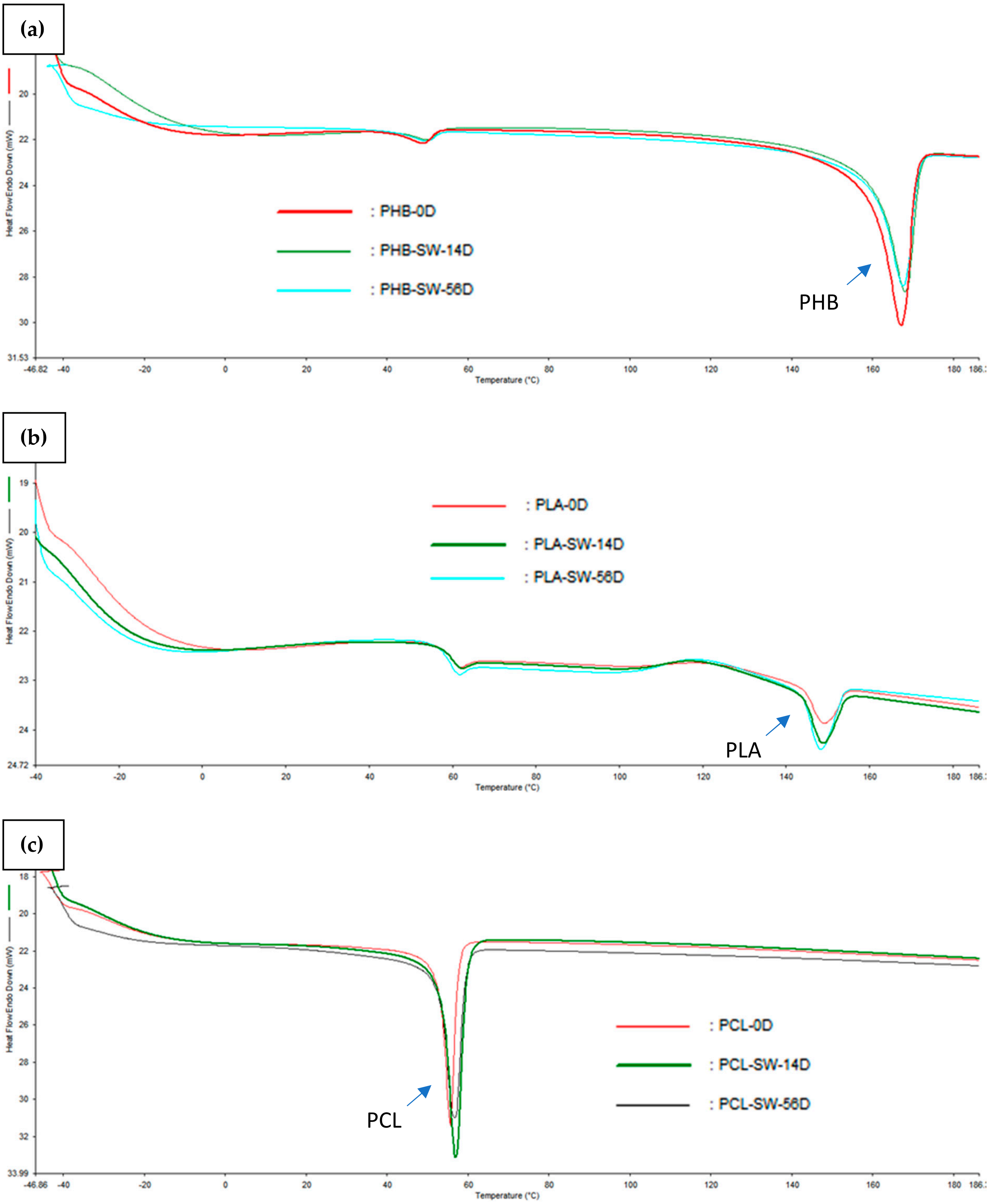

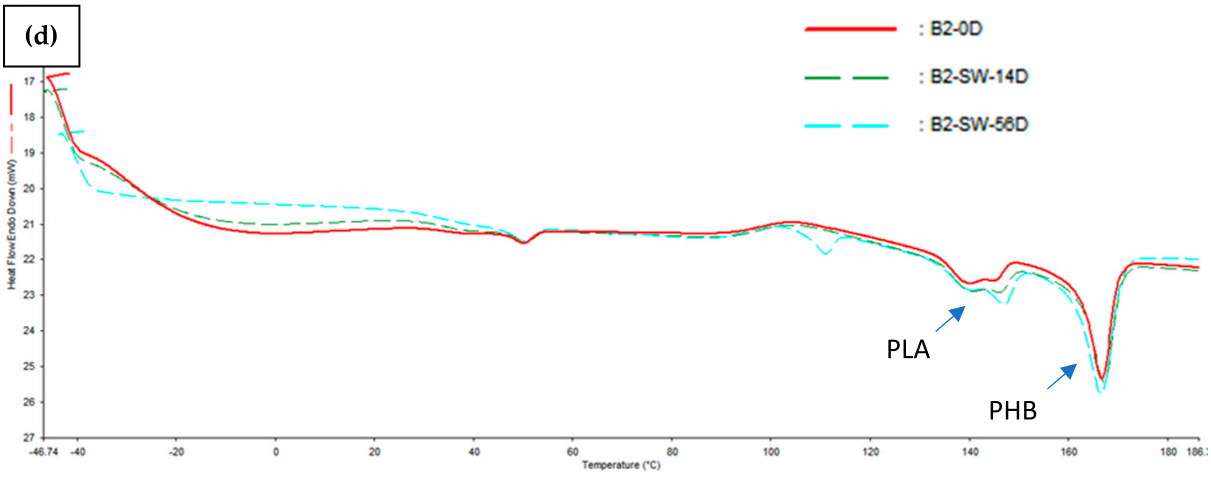
(d)
: B2-0D
: B2-SW-14D
: B2-SW-56D
Heat FlowEndo Down (mW)
Temperature (°C)
PLA
PHB

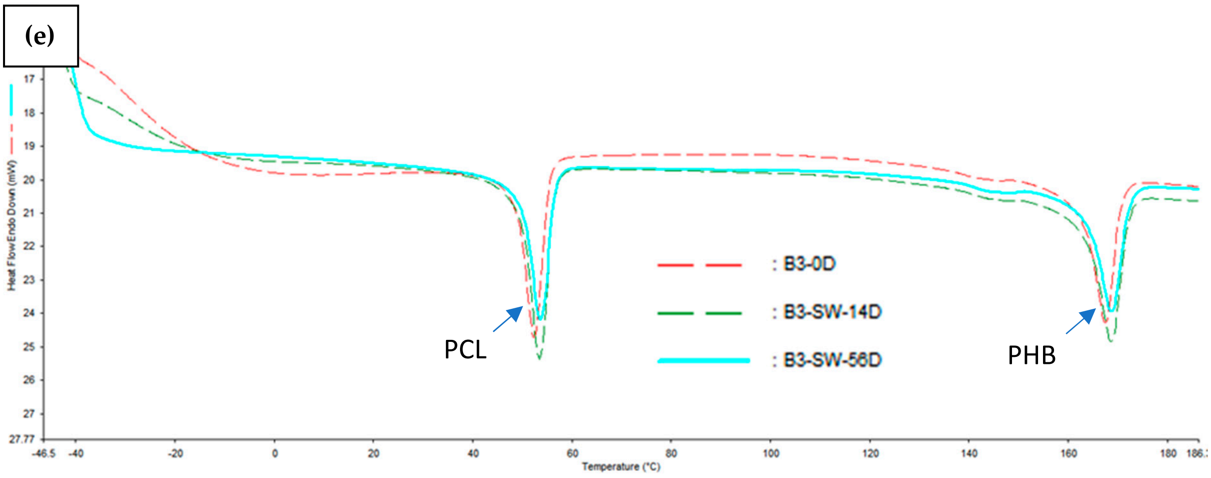
(e)
: B3-0D
: B3-SW-14D
: B3-SW-56D
Heat FlowEndo Down (mW)
Temperature (°C)
PCL
PHB

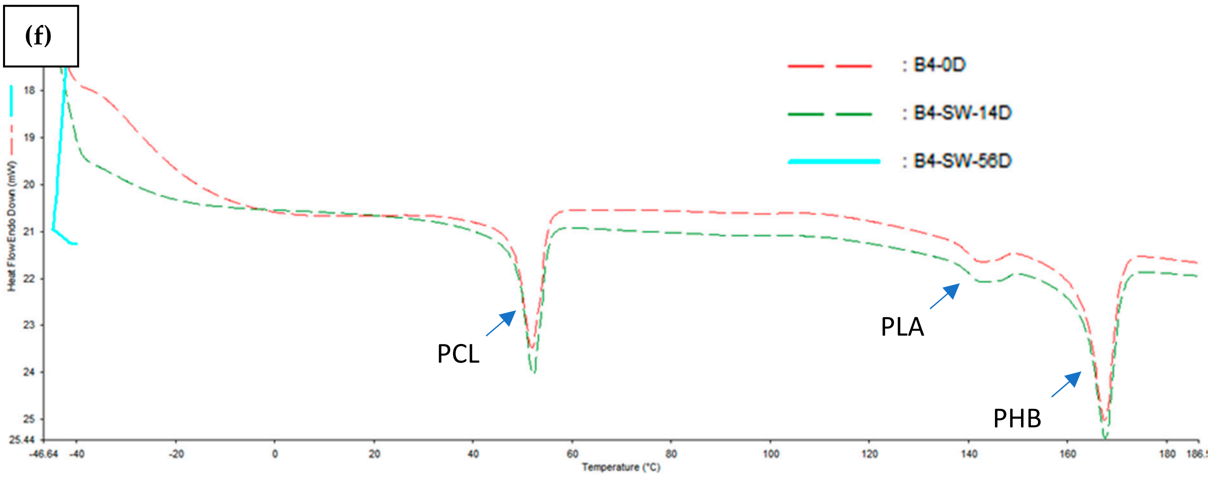
(f)
: B4-0D
: B4-SW-14D
: B4-SW-56D
Heat FlowEndo Down (mW)
Temperature (°C)
PCL
PLA
PHB

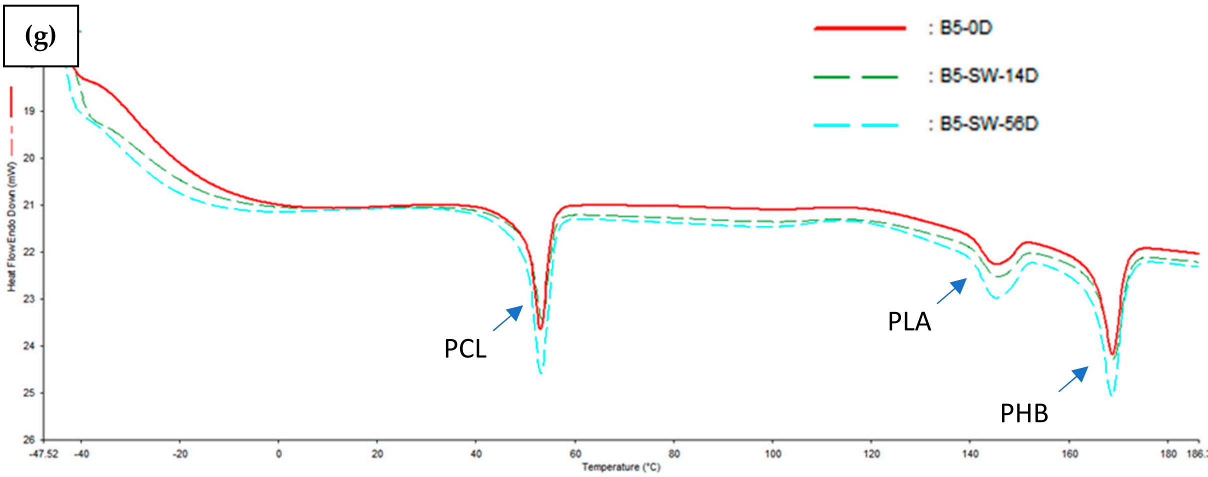

Figure S4. DSC of neat polymers PHB/PLA/PCL and their respective blends: (**a**) PHB, (**b**) PLA, (**c**) PCL, (**d**) B2: 50/50/0, (**e**) B3: 50/0/50, (**f**) B4: 50/25/25, and (**g**) B5: 30/50/20 (wt. % PHB/PLA/PCL).
